# Feasibility of using a dual-promoter recombinant baculovirus vector to coexpress EGFP and GDNF in mammalian cells

**DOI:** 10.3892/etm.2014.1655

**Published:** 2014-03-31

**Authors:** JIANZHANG WANG, JUN WANG, CHANGPING CAI, SHILI WANG, SHUAI LIU, SHUO SHI, YIFAN ZHANG, BIAO LI

**Affiliations:** 1 Department of Otolaryngology, Rui Jin Hospital, Shanghai Jiao Tong University School of Medicine, Shanghai 200025, P.R. China; 2 Department of Nuclear Medicine, Rui Jin Hospital, Shanghai Jiao Tong University School of Medicine, Shanghai 200025, P.R. China

**Keywords:** baculovirus, dual-promoter, coexpression, gene delivery vector, dual-gene delivery

## Abstract

Vectors that are capable of coexpressing two or more exogenous genes for *in vitro* and *in vivo* gene delivery are being increasingly studied. The aim of the present study was to explore the feasibility of using the pFastBac™ Dual vector, under the control of two cytomegalovirus (CMV) promoters with opposite directions, to coexpress enhanced green fluorescent protein (EGFP) and glial cell line-derived neurotrophic factor (GDNF) in the same mammalian cell. In the study, two promoters in the pFastBac Dual vector were replaced with CMV-EGFP and CMV-GDNF, whose directions were consistent with the initial directions. The pFastBac Dual-CMV-EGFP-CMV-GDNF plasmid was constructed and then transfected into human embryonic kidney (HEK) 293T cells. The recombinant virus, Bac Dual-CMV-EGFP-CMV-GDNF, was generated with the Bac-to-Bac Baculovirus Expression system and used to transduce HeLa cells. Immunofluorescence was applied to examine the coexpression of EGFP and GDNF in transfected or transduced mammalian cells, while western blot analysis was used to confirm the expression of GDNF in transduced HeLa cells. The recombinant plasmid was constructed and the recombinant baculovirus was successfully generated. Immunofluorescence observations demonstrated that EGFP and GDNF were simultaneously expressed in the same transfected HEK 293T cell and in a single transduced HeLa cell. Western blot analysis revealed that GDNF was expressed accurately in the transduced cells. Therefore, the pFastBac Dual vector is an efficient gene transfer vector that is able to coexpress two target proteins in mammalian cells and serve as a platform for combining reporter or/and therapy genes used in molecular imaging and dual-gene therapy. Thus, the current study presents a new coexpression strategy for dual-gene delivery *in vitro* and *in vivo*.

## Introduction

Gene therapy has been defined as the deliberate introduction of genetic material into cells in order to treat or prevent a disease. The success of gene therapy ultimately depends on gene delivery vectors. Thus, a number of studies have focused on creating more efficient, stable and safe gene therapy vectors. Viral and non-viral vectors are the two main groups of vectors that are used in gene therapy. To date, ~70% of clinical trials and the majority of studies have used viral vectors. Widely used viral vectors include adenovirus, adeno-associated virus, retrovirus and lentivirus. However, these vectors possess limitations, including possible safety problems (1,2) and challenges for large scale production. In contrast to the commonly employed viral vectors, baculovirus has unique properties of inherent safety, ease and speed of virus generation in high quantities, low cytotoxicity and extreme transgene capacity (3). Thus, the baculovirus vector is being increasingly applied and continually developed for efficient gene therapy.

The baculovirus expression vector system (BEVS) is recognized as a feasible and safe technology to produce recombinant proteins in insect cells, which are eukaryotic. The basis of BEVS lies in large enveloped DNA viruses derived from insects. The prototype virus is *Autographa californica* multiple nucleopolyhedrovirus (AcMNPV), which uses a 134-kbp genome to encode 156 viral proteins. AcMNPV is occluded in a polyhedrin (PH) and p10 proteins and expression of the virus is under the control of PH and p10 promoters, respectively. However, as these promoters are not indispensable for viral replication, the PH or p10 promoter may be replaced by other promoters. Infection of insect cells with a virus encoding a desired transgene under the control of the powerful baculovirus PH or p10 promoter leads to recombinant protein production in high quantities (4). In 1995, it was demonstrated that a recombinant baculovirus was able to infect mammalian cells and express foreign genes under the control of a mammalian promoter (5). Since then, numerous cells have been shown to be permissive for baculovirus-mediated gene delivery, including animal cells from humans, cows, rodents, pigs, rabbits, fish or with an avian origin, as well as a number of primary cells, including embryonic, adult and induced pluripotent stem cells (6).

The Bac-to-Bac Baculovirus Expression system (Invitrogen Life Technologies, Carlsbad, CA, USA) provides a rapid and efficient method for generating recombinant baculoviruses (7). A number of pFastBac™ vectors, including pFastBac 1, HT and Dual, may be used in this system. The pFastBac 1 and HT vectors have a strong PH promoter for high-level protein expression, while the pFastBac Dual vector is a non-fusion vector that has two strong baculovirus promoters, PH and p10, which allow the simultaneous expression of two proteins. Previously, PH or p10 promoters have been replaced by mammalian promoters in one of the aforementioned pFastBac vectors. However, to date, to the best of our knowledge, there have been no studies in which the PH and p10 promoters in the pFastBac Dual vector have been replaced with mammalian promoters. Originally, the pFastBac Dual vector allowed the production of two proteins in insect cells only. The aim of the present study was to investigate the effect of replacing the two promoters with two strong mammalian promoters and to determine whether the vector was effective in coexpressing two target proteins in mammalian cells. If successful, this would expand the application of the vector from protein expression in insect cells to dual-gene delivery *in vitro* and *in vivo*.

Coexpression of two or more exogenous genes in an organism becomes increasingly significant with genetic engineering since the metabolic pathways and physiological processes are complex and require the coordination functions of a variety of genes. Currently, widely used multiexpression strategies include coinfection (8), internal ribosome entry sites (IRES) (9), fusion protein (10) and a self-cleaving 2A peptide (11). However, these strategies have disadvantages. Firstly, the correlation between the expression levels of the two genes is usually hard to examine in the coinfection approach. Secondly, IRES elements can be large and attenuate the expression of downstream genes. Thirdly, fusion protein production may result in a compromised function, which may potentially be due to improper protein folding or trafficking. Finally, cloning vectors harboring a 2A peptide gene are not publicly available. One method to overcome these problems is the use of a dual-expression vector that includes two promoters. In addition, multi-gene therapy is an inevitable trend in gene therapy development as it provides a more rational and superior approach compared with single-gene therapy. Due to the advantages of the Bac-to-Bac Baculovirus Expression system, the pFastBac Dual vector was selected for the current study. However, this vector can only express proteins in insect cells. Thus, in the present study, a pFastBac Dual vector was developed that possessed a gene cassette consisting of the enhanced green fluorescent protein (EGFP) gene and glial cell line-derived neurotrophic factor (GDNF) gene. The two genes were under the control of the cytomegalovirus (CMV) promoter and generated a recombinant baculovirus named Bac Dual-CMV-EGFP-CMV-GDNF. Human embryonic kidney (HEK) 293T cells and HeLa cells were transduced with this recombinant baculovirus. Indirect immunofluorescence was applied to demonstrate EGFP and GDNF expression in a single cell simultaneously and western blot analysis was performed to detect the expression of the protein of interest. Therefore, the present study focused on developing a dual-promoter and coexpressing vector.

## Materials and methods

### Main reagents

**Figure 1:**
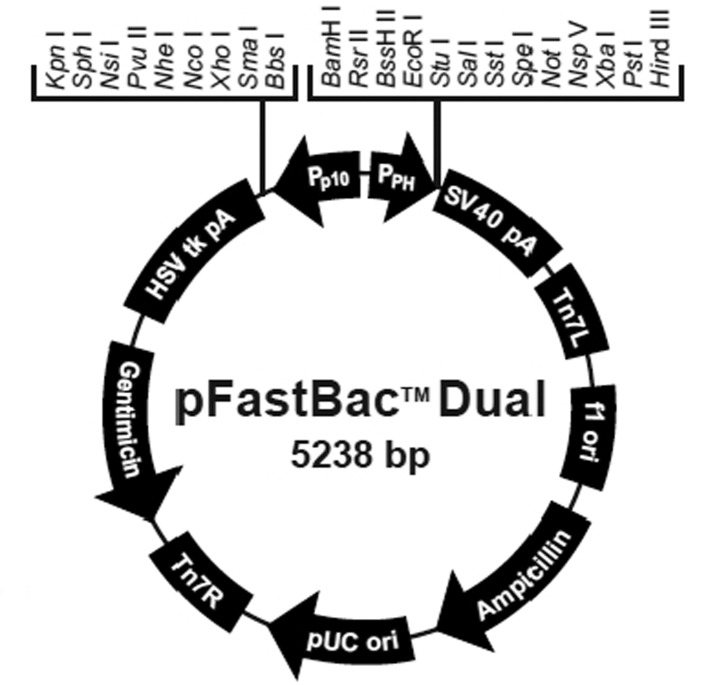
Map of the pFastBac Dual vector.

The pFastBac Dual vector (Fig. 1), DH10Bac *Escherichia coli (E. coli)* cells, Sf9 cells, PureLink HiPure Plasmid Midiprep kit, Lipofectamine 2000, Cellfectin II reagent, fetal bovine serum (FBS), Dulbecco’s modified Eagle’s medium (DMEM) and Sf-900 III serum-free medium were purchased from Invitrogen Life Technologies (Shanghai, China). DH5α *E. coli* competent cells, restriction endonuclease, T4 DNA ligase, LA *Taq* with GC buffer and DNA markers were products of Takara Bio, Inc. (Dalian, China). DNA extraction and plasmid DNA purification kits were obtained from Qiagen (Germantown, MD, USA). Rat GDNF and pEGFP-1 plasmids were conserved in the laboratory at Rui Jin Hospital (Shanghai, China). Anti-GDNF rabbit polyclonal antibodies were purchased from Santa Cruz Biotechnologies, Inc. (Santa Cruz, CA, USA). DyLight594 goat anti-rabbit IgG was purchased from MultiSciences Biotech Co., Ltd. (Hangzhou, China). Horseradish peroxidase was purchased from Beyotime Institute of Biotechnology (Shanghai, China). Primers were synthesized and DNA was sequenced by Invitrogen Life Technologies (Shanghai, China).

### Cell lines and culture

Sf9 cells were propagated at 27°C in Sf-900 III serum-free medium. HeLa and HEK 293T cells, provided by the Cell Bank of the Chinese Academy of Science (Shanghai, China), were cultured in DMEM supplemented with 10% FBS in a humidified environment with 5% CO_2_ at 37°C.

### Construction of the pFastBac Dual-CMV-EGFP-CMV-GDNF plasmid

Polymerase chain reaction (PCR) primers, F1 and R1, were designed for CMV-EGFP (Table I). The *Bam*HI gene (GGATCC) was added to the 5′ end of primer F1, while the *Kpn*I gene (GGTACC) and terminator codon (TTA) were added to the 5′ end of the R1 primer. Next, a CMV-EGFP fragment was amplified from the pEGFP-C1 vector by PCR with the F1 and R1 primers. The CMV-EGFP fragment and pFastBac Dual vector were digested simultaneously with *Bam*HI and *Kpn*I and then connected with T4 DNA ligase following electrophoresis and recovery. *E. coli* DH5α cells were transformed with 5 μl connected products. A single colony was selected and the pFastBac Dual-CMV-EGFP plasmid was obtained following verification by gene sequencing.

PCR splicing primers, F2, R2, F3 and R3, were designed for CMV-GDNF (Table I). The *Sal*I gene (GTCGAC) was added to the 5′ end of primer F2 and the *Hin*dIII gene (AAGCTT) was added to the 5′ end of primer R3. The CMV fragment was amplified from the pEGFP-C1 vector by PCR with the F2 and R2 primers, while the GDNF fragment was amplified from the GDNF plasmid with the F3 and R3 primers. Next, the CMV and GDNF fragments were connected by overlap extension PCR with the F2 and R3 primers and the CMV-GDNF fragment was obtained following the recovery of the PCR products.

The pFastBac Dual-CMV-EGFP vector and CMV-GDNF fragment were digested simultaneously with *Sal*I and *Hin*dIII and connected with T4 DNA ligase following electrophoresis and recovery. Next, 5 μl connected products was transformed into *E. coli* DH5α cells. A single colony was selected and the pFastBac Dual-CMV-EGFP-CMV-GDNF plasmid was obtained following verification by 1.2% agarose gel electrophoresis and sequencing.

### Generation of recombinant baculovirus

Following the construction of the pFastBac Dual-CMV-EGFP-CMV-GDNF plasmid, it was transformed into DH10Bac *E. coli* cells for transposition into a bacmid. A white colony and a blue colony were selected by blue/white colony selection and the PureLink HiPure Plasmid Midiprep kit was used to purify recombinant bacmid DNA. To verify the presence of the gene of interest in the recombinant bacmid from the white colony, the specific, pUC/M13 forward and reverse primers were synthesized (Table I). PCR was then performed with pUC/M13 primers or with the pUC/M13 forward and specific primers. Correspondingly, PCR was performed with pUC/M13 primers and the bacmid from the blue colony. The obtained reaction products were analyzed by agarose gel electrophoresis.

Transfection of Sf9 cells with the recombinant bacmid and Cellfectin II was performed according to the manufacturer’s instructions. Cell morphology was observed daily to view the characteristics of viral infection following transfection. After 7 days, the supernatant was collected and the P1 viral stock was obtained. The P2 viral stock was obtained following the amplification of the P1 viral stock. To determine the titer of the baculoviral stock, a viral plaque assay was performed.

### Transfection of HEK 293T cells with Lipofectamine 2000

One day prior to transfection, HEK 293T cells were plated in a 24-well plate coated with poly-L-lysine in DMEM without antibiotics with the result that the cells were 80–90% confluent at the time of transfection. Next, the recombinant plasmid was transfected into HEK 293T cells with Lipofectamine 2000, according to the manufacturer’s instructions, to prepare for immunofluorescence.

### Transduction of HeLa cells with recombinant baculovirus

HeLa cells were seeded in a 24- or 6-well plate with DMEM and 10% FBS for 24 h. The medium was replaced with phosphate-buffered saline (PBS) just prior to virus transduction. Recombinant baculovirus was then added at a multiplicity of infection of 200. Cells treated with PBS only were used as a negative control. Cells were incubated at 37°C for 4 h following treatment and the medium, including the virus, was replaced with DMEM and 10% FBS. After 24 h, the cells in the 24- or 6-well plates were analyzed by immunofluorescence and western blotting.

### Immunofluorescence test

Following the transfection of HEK 293T cells with Lipofectamine 2000 and the transduction of HeLa cells in a 24-well plate with recombinant baculovirus, an indirect immunofluorescence test was performed with a 1:300 dilution of anti-GDNF rabbit polyclonal antibodies and a 1:300 dilution of DyLight594 goat anti-rabbit IgG. The cells were observed with a fluorescence microscope (Olympus Corporation, Tokyo, Japan).

### Western blot analysis

Following the transduction of HeLa cells in a 6-well plate with recombinant baculovirus, the cells were collected and protein extracts were prepared in a lysis buffer. Western blot analysis was then performed by incubating the filtrate with a 1:500 dilution of anti-GDNF rabbit polyclonal antibodies in Tris-buffered saline Tween 20 at 4°C overnight. Next, a 1:1,000 dilution of secondary antibodies conjugated to horseradish peroxidase was added for 1 h at room temperature. Protein bands were treated using an enhanced chemiluminescence assay kit (PerkinElmer, Inc., Waltham, MA, USA).

## Results

### Verification of the recombinant plasmid

**Figure 2:**
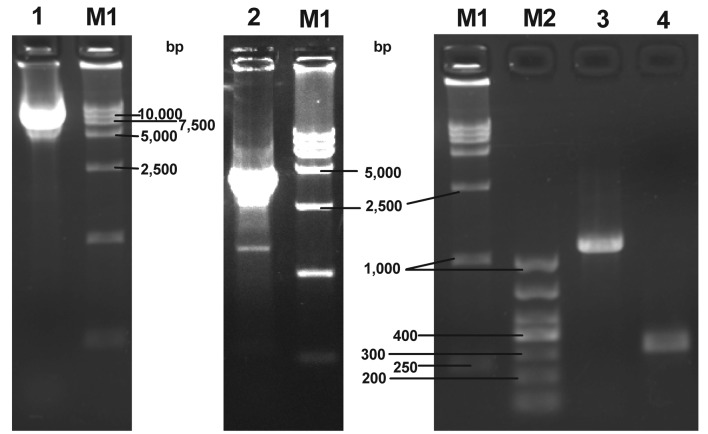
Agarose gel electrophoresis of the recombinant plasmid and bacmid DNA PCR products. Lane 1, pFastBac Dual-CMV-EGFP-CMV-GDNF plasmid; lane 2, PCR product of recombinant bacmid DNA amplified with pUC/M13 forward and reverse primers; lane 3, PCR product of recombinant bacmid DNA amplified with the specific and pUC/M13 reverse primers; lane 4, PCR product of non-recombinant bacmid DNA amplified with pUC/M13 forward and reverse primers; lane M1, DL 15,000 DNA marker; lane M2, DL 1,000 DNA marker; PCR, polymerase chain reaction; CMV, cytomegalovirus; EGFP, enhanced green fluorescent protein; GDNF, glial cell line-derived neurotrophic factor.

Following the construction of the pFastBac Dual-CMV-EGFP-CMV-GDNF plasmid, the size was evaluated by 1.2% agarose gel electrophoresis. As shown in Fig. 2, the electrophoresis stripe in lane 1 was at the correct site (~7,426 bp). In addition, the sequencing results demonstrated that the inserted gene fragment was in accordance with expectations and there was no mutation (data not shown). Therefore, the pFastBac Dual-CMV-EGFP-CMV-GDNF plasmid was constructed successfully.

### Generation and confirmation of the recombinant bacmid

As shown in lanes 2 and 3 of Fig. 2, PCR products of the expected sizes were obtained (~4,800 bp and 1,200 bp) from the white colony, indicating the presence of the gene of interest in the recombinant bacmid. pUC/M13 forward and reverse primers were selected to amplify the bacmid DNA from the blue colony and a PCR product of the expected size (~300 bp) was achieved, as shown in lane 4 (Fig. 2). This demonstrated that no transposition occurred in the blue colony. Thus, the results indicated that transposition occurred in the white colony and the expected recombinant bacmid was isolated.

### Generation of the recombinant baculovirus

Following transfection with Cellfectin II, Sf9 cells typically exhibited characteristics of infected cells emerging in succession as follows: Increased cell diameter, increased nuclei size, cessation of cell growth, granular appearance, detachment and cell lysis. These qualities demonstrated that Bac Dual-CMV-EGFP-CMV-GDNF was constructed successfully. In addition, the viral plaque assay revealed that the titer of the baculoviral stock was 8×10^7^ pfu/ml.

### Identification by immunofluorescence

**Figure 3:**
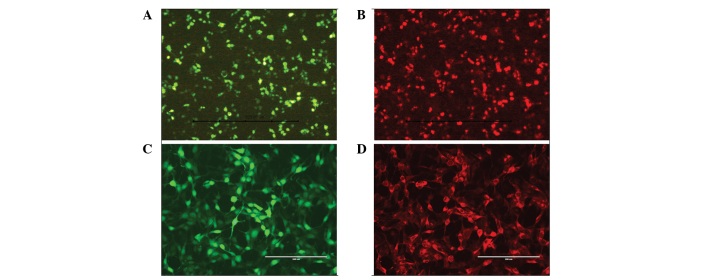
Confirmation of EGFP and GDNF coexpression in transfected HEK 293T cells and transduced HeLa cells. (A) Green fluorescence from EGFP and (B) red fluorescence immunostained by anti-GDNF antibody in HEK 293T cells transfected with the recombinant plasmid and Lipofectamine 2000. (C) Green fluorescence from EGFP and (D) red fluorescence immunostained by anti-GDNF antibody in HeLa cells transduced by the recombinant baculovirus. EGFP, enhanced green fluorescent protein; GDNF, glial cell line-derived neurotrophic factor; HEK, human embryonic kidney.

Immunofluorescence analysis indicated that EGFP and GDNF were simultaneously expressed in the same HEK 293T cell transfected with recombinant plasmid and Lipofectamine 2000 (Fig. 3A and B), confirming the accuracy and usability of the recombinant plasmid. The synchronous expression of EGFP and GDNF in a single HeLa cell transduced by recombinant baculovirus demonstrated that the pFastBac Dual vector, under the control of two mammalian promoters, was able to coexpress two target genes in mammalian cells (Fig. 3C and D).

### Western blot analysis

**Figure 4:**
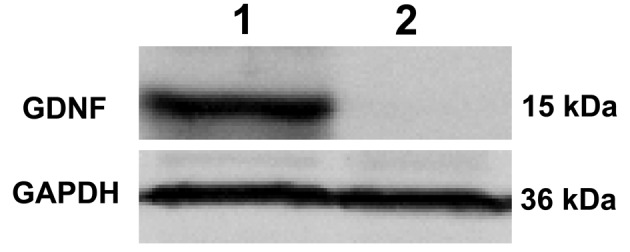
Western blot analysis of GDNF expression in HeLa cells transduced with the recombinant baculovirus. Lane 1, infected cell lysate; lane 2, non-infected cell lysate; GDNF, glial cell line-derived neurotrophic factor.

As shown in Fig 4, western blot analysis results revealed no protein band in lane 2. However, a distinct protein band with a relative molecular weight of ~15 kDa corresponding to GDNF was observed in lane 1 (Fig. 4). This confirmed that GDNF was not expressed in the non-transduced HeLa cells, while GDNF was expressed in the HeLa cells transduced by recombinant baculovirus. Therefore, western blot analysis demonstrated that GDNF was expressed accurately in cells transduced with Bac-CMV-EGFP-CMV-GDNF.

## Discussion

GDNF was first identified as a potent survival factor for midbrain dopaminergic neurons, but was then shown to be a potent neurotrophic factor that had restorative effects in a wide variety of rodent and primate models of Parkinson’s disease (12). GDNF is broadly expressed and essential for the development of the kidney and the enteric nervous system. As a multifunctional protein, GDNF has the ability to induce cellular survival, proliferation, migration and differentiation (13). To date, the scope of GDNF applications has been greatly expanded and includes the treatment of Huntington’s disease, amyotrophic lateral sclerosis, chronic pain, depression and addiction, as well as the regeneration of the sciatic nerve (14). In view of its numerous applications, GDNF was selected as the target protein in the present study.

In this study, the PH and p10 promoters in the pFastBac Dual vector were replaced by CMV promoters, whose directions were consistent with the initial direction of the PH or p10 promoter. A CMV-EGFP gene fragment was inserted into the initial region of the p10 promoter gene and a CMV-GDNF gene fragment, connected by the CMV promoter and GDNF genes, was cloned into the initial site of the PH promoter gene. Following the successful construction of the pFastBac Dual-CMV-EGFP-CMV-GDNF plasmid, it was transfected into HEK 293T cells with Lipofectamine 2000. Through indirect fluoroimmunoassay technology, EGFP and GDNF were shown to be expressed in a single HEK 293T cell at the same time, which confirmed the accuracy and usability of the recombinant plasmid. Next, Bac Dual-CMV-EGFP-CMV-GDNF was generated using the Bac-to-Bac Baculovirus Expression system. A viral plaque assay demonstrated that the virus titer was 8×10^7^ pfu/ml. HeLa cells were transduced with the recombinant baculovirus. Similarly, the simultaneous existence of EGFP and GDNF in the same HeLa cell was demonstrated by fluorescence detection and GDNF protein was identified by western blot analysis. Therefore, in the present study, a dual-promoter recombinant baculovirus vector was constructed and shown to coexpress EGFP and GDNF in mammalian cells.

Considering that GDNF must exist when EGFP is expressed in the gene expression of Bac-CMV-EGFP-CMV-GDNF, the position and quantity of expressed GDNF can be conveniently estimated through observing the expression of EGFP. If the EGFP gene were to be replaced with an imaging reporter gene, non-invasive imaging of the reporter gene products would reveal the temporal and spatial biodistribution of GDNF or other genes of interest in virtually any location within living subjects. Molecular imaging of reporters for target genes plays a vital role in optimizing gene therapy by quantitatively imaging reporter gene expression and the therapeutic effect of transgenes *in vivo*. The sodium iodide symporter (NIS) has emerged as one of the most promising reporter genes in preclinical and translational studies (15). Thus, combining molecular imaging and gene therapy is likely to be conducive in enhancing the efficacy and safety of the current gene therapy protocols for human application and in supporting future individualized patient treatment (16). In addition, stem cell studies may require the coexpression of two marker genes, one driven by a constitutive promoter to monitor gene transfer or track the modified cells and the other driven by a lineage-specific promoter to monitor stem cell differentiation. In addition, the transduction efficiency of baculovirus is quite high in mesenchymal stem cells (17). Therefore, the dual-promoter recombinant baculovirus vector significantly facilitates the monitoring of gene delivery *in vitro* and *in vivo*.

With regard to therapy, certain therapeutic applications require the coexpression of multiple genes in the same cell. Specific applications may require the generation of several proteins that function synergistically as part of a network or produce multiple subunits of a protein that are expressed by various genes. There is increasing evidence that effective treatments for specific diseases may demand the expression of multiple therapeutic proteins that are selected to treat specific aspects of the disease process. For example, an Ad5 viral vector coexpressing human thrombopoietin (hTPO) and human NIS proteins in tumor cells (Ad-CMV-hTPO-T2A-hNIS) enhanced radioiodine uptake and prolonged radioiodine retention (18). Furthermore, brain-derived neurotrophic factor (BDNF) and neurotrophin-3 (NT3) coexpression using a glucocorticoid (GC)-induced bicistronic expression vector (pGC-BDNF-IRES-NT3) protected apoptotic cells in a cellular injury model (19). One study generated 2A-harboring cloning vectors that were likely to be useful for bicistronic or multicistronic expression (11). In another study, a fusion suicide gene was combined with human telomerase reverse transcriptase (hTERT)-targeted shRNA in a new combined plasmid to provide an antitumor effect via the synergistic actions of suicide gene therapy and the targeting of hTERT through RNAi (20). However, each strategy has its own properties and shortcomings, as aforementioned. In the present study, in view of the advantages of the Bac-to-Bac Baculovirus Expression system, the pFastBac Dual vector was reconstructed with primary success.

The pFastBac Dual vector contains two multiple cloning sites to allow the expression of two heterologous genes. One gene is controlled by the PH promoter and the other by the p10 promoter, resulting in the production of non-fusion proteins. Molecular cloning technology can be applied to clone interest gene(s) into the pFastBac Dual vector. An ATG start codon for the initiation of translation and a stop codon for the termination of the gene must be contained in the inserts to ensure proper expression of recombinant proteins. Once the inserts are cloned into the pFastBac Dual vector, transformation into DH10Bac *E. coli* cells is performed to generate a recombinant bacmid, which is later transfected into Sf9 cells. Thus, a recombinant baculovirus is generated following transfection. The Bac-to-Bac Baculovirus Expression system facilitates the rapid and efficient generation of a recombinant baculovirus.

In the present study, Bac Dual-CMV-EGFP-CMV-GDNF was successfully produced and the pFastBac Dual vector was demonstrated to be an efficient gene transfer vector, fulfilling the strategy of double gene coexpression. Due to highly efficient gene delivery by baculoviruses, there have been major advances in the application of baculoviruses in molecular imaging and gene therapy. Previously, the most commonly used method for baculovirus-mediated gene imaging was the coinfection approach, where one vector encodes a gene of interest and an additional vector encodes a reporter gene. Now, the reconstructed pFastBac Dual vector is a potential alternative for gene imaging. In addition, the applications of the pFastBac Dual vector may be markedly expanded in gene therapy, particularly in cases requiring dual gene-coordination.

In conclusion, the pFastBac Dual vector with two promoters of opposite directions was an efficient gene transfer vector that coexpressed two target genes and served as a platform for combining reporter or/and therapy genes. Therefore, a novel and feasible method of coexpression with dual-promoters has been proposed in the present study. The pFastBac Dual vector is likely to yield numerous advantages in the future.

## Figures and Tables

**Table I:** Primers used for the amplification of CMV-EGFP and CMV-GDNF and the analysis of recombinant bacmid by PCR.

Primer name	Sequence, 5′→3′
F1	GCCGCCGGATCCTAGTTATTAATAGTAATCAATTACGGGGTCA
R1	ACCACCGGTACCTTACTTGTACAGCTCGTCCATGCC
F2	GCCGCCGTCGACTAGTTATTAATAGTAATCAATTACGGGGTCA
R2	CGACATCCCATAACTTCATGGTGGCGATCTGACGGTTCACTAAACCAGCT
F3	TTTAGTGAACCGTCAGATCGCCACCATGAAGTTATGGGATGTCGTGGCT
R3	ACCACCAAGCTTTCAGATACATCCACACCGTTTAGC
pUC/M13 forward	CCCAGTCACGACGTTGTAAAACG
pUC/M13 reverse	AGCGGATAACAATTTCACACAGG
Specific primer	ATGAAGTTATGGGATGTCGTGG

CMV, cytomegalovirus; EGFP, enhanced green fluorescent protein; GDNF, glial cell line-derived neurotrophic factor; PCR, polymerase chain reaction.
